# Targeting KRAS^G12C^ in non-small cell lung cancer: evidence from randomized clinical trials and future therapeutic strategies

**DOI:** 10.3389/fonc.2026.1916211

**Published:** 2026-08-19

**Authors:** Julia Piekarz, Natalia Picheta, Jakub Pobideł, Natalia Gierulska, Karolina Kłodnicka, Jacek Januszewski, Katarzyna Szklener, Magdalena Skórzewska

**Affiliations:** 1 Student Academic Group, Department of Clinical Oncology and Chemotherapy, Medical University, Lublin, Poland; 2 Doctoral School, Medical University of Lublin, Lublin, Poland; 3 Department of Clinical Oncology and Chemotherapy, Medical University, Lublin, Poland

**Keywords:** adagrasib, KRAS mutation, KRAS^G12C^, NSCLC, sotorasib

## Abstract

**Background:**

The Kirsten rat sarcoma viral oncogene homolog ^G12C^ (KRAS^G12C^) mutation is no longer an “elusive” target, having become an important therapeutic target in non-small cell lung cancer (NSCLC). The approvals of sotorasib and adagrasib have significantly altered the second-line treatment algorithm. This systematic review critically evaluates and compares data from Phase III clinical trials and the mechanisms of resistance associated with these two inhibitors.

**Methods:**

The systematic review was registered in the PROSPERO database and conducted in accordance with the Preferred Reporting Items for Systematic Reviews and Meta-Analyses (PRISMA) 2020 guidelines. PubMed, Scopus, and ClinicalTrials.gov were searched for phase III randomized controlled trials (RCTs) conducted between 2023 and 2026. The analysis included the CodeBreaK 200 and KRYSTAL-12 registration programs (including subanalyses), which evaluated monotherapy with sotorasib or adagrasib compared with docetaxel in patients with advanced NSCLC harboring the KRAS^G12C^ mutation. The risk of bias was assessed using the Cochrane RoB 2.0 tool.

**Results:**

Both inhibitors significantly prolonged the median progression-free survival (PFS) and increased the objective response rate (ORR) compared with chemotherapy. Adagrasib demonstrated promising intracranial efficacy but was associated with greater gastrointestinal toxicity and frequent dose reductions. Sotorasib was characterized by a better safety profile and more favorable quality-of-life outcomes for patients, although the assessment of its impact on overall survival (OS) was confounded by a high crossover rate in the registration trial. However, the rapid development of secondary mutations and bypass resistance mechanisms remains the main clinical limitation of both drugs when used as monotherapy.

**Conclusion:**

Sotorasib and adagrasib provide clinical benefit in advanced NSCLC with the KRAS^G12C^ mutation, demonstrated improved clinical outcomes compared with docetaxel monotherapy. However, to overcome the barrier of inevitable biological resistance, it is crucial to seek new therapeutic approaches and intensively develop combination strategies based on these molecules, which supports the ongoing development of combination strategies in personalized oncology.

**Systematic review registration:**

https://www.crd.york.ac.uk/prospero/, identifier CRD420261427962.

## 1 Introduction

Lung cancer remains one of the deadliest cancers in the world, accounting for the majority of cancer-related deaths. According to global epidemiological data from the Global Cancer Observatory 2022 (GLOBOCAN 2022), this cancer was the most commonly diagnosed neoplastic disease worldwide, accounting for nearly 2.5 million new cases (12.4% of all cases) and approximately 1.8 million deaths annually (1). Histologically, it is divided into non-small cell lung cancer (NSCLC), accounting for approximately 85% of cases, and small cell lung cancer (SCLC). Within NSCLC, the adenocarcinoma subtype plays a key role, as it is characterized by the highest frequency of driver mutations (2).

Modern diagnosis and treatment of NSCLC have undergone a profound evolution. A key factor in improving prognosis has been the implementation of low-dose computed tomography (LDCT) in screening, which allows for the detection of tumors at a fully curable stage. A pivotal trend at present is the shift toward using immunotherapy and tyrosine kinase inhibitors (TKIs) for neoadjuvant (preoperative) and perioperative treatment in stages IB–III, which substantially reduces the previously high (30–75%) risk of postoperative recurrence (3, 4).

In advanced-stage disease, traditional chemotherapy has been replaced by dual immune checkpoint inhibition based on anti-programmed cell death protein 1/programmed death-ligand 1 (anti-PD-1/PD-L1) combined with anti-cytotoxic T-lymphocyte-associated antigen 4 (anti-CTLA-4) in patients without driver mutations, as well as by the rapidly evolving platform of antibody-drug conjugates (ADCs) and highly selective next-generation TKIs (2, 5). This rapid advancement in precision medicine and modern clinical meta-analyses has made it possible to directly target second-line treatment—the former, conventional docetaxel-based standard of care is now being replaced by targeted therapies with more favorable toxicity profiles and improved tolerability (5, 6).

In the pathophysiology of adenocarcinoma of NSCLC, driver mutations play a key role, as they permanently render the cancer cell dependent on growth signals (7). This pathogenesis is most commonly driven by the Kirsten rat sarcoma viral oncogene homolog (KRAS) gene, which is the most common oncogene detected in approximately 25% of patients with lung adenocarcinoma; in Europe, this rate reaches as high as 30%, while in Asian and Latin American countries it is significantly lower. One of the most important alterations is the KRAS^G12C^ mutation, the prevalence of which shows significant geographical differences: it is reported in 9.3–18.4% of patients in Europe and 8.9–19.5% in the U.S., while in Latin America it ranges from 6.9–9.0%, and in Asia, as low as 1.4–4.3% (8, 9).

Physiologically, the KRAS protein functions as an intracellular molecular switch within the mitogen-activated protein kinase (MAPK) pathway, oscillating between an inactive (guanosine diphosphate =GDP-bound) and an active (guanosine triphosphate=GTP-bound) state. However, the somatic G12C point mutation (a glycine-to-cysteine substitution at codon 12) locks the protein in a permanent, active “ON” state (10, 11). This results in continuous, autonomous stimulation of cell proliferation, rapid tumor progression, and resistance to conventional therapies. Crucially for prognosis, KRAS^G12C^-mutant tumors exhibit high genomic heterogeneity and frequently co-occur with other key mutations, such as *tumor protein p53 (TP53) (17.8–50%), serine/threonine kinase 11 (STK11) (10.3–28%), and kelch-like ECH-associated protein 1 (KEAP1) (6.3–23%)* (8).

For more than four decades, the KRAS protein was considered a completely pharmacologically inaccessible target due to the lack of deep binding pockets on its surface. A structural milestone occurred in 2013 with the discovery by Prof. Kevin Shokat’s team of a new, hidden allosteric pocket, Switch-II (SII-P) (11). It was demonstrated that small molecules can covalently and irreversibly bind to this niche structure, stabilizing the protein in its inactive GDP-bound conformation (OFF state) and thereby interrupting the oncogenic signaling cascade (12).

This pivotal discovery led to rapid advances in medicinal chemistry and resulted in the synthesis of the first small-molecule inhibitors of their kind. Based on landmark clinical trial results, the U.S. Food and Drug Administration (FDA) granted accelerated approval to two leading compounds: sotorasib (AMG 510) was approved in May 2021, and adagrasib (MRTX849) was approved in December 2022 (13, 14). Both of these drugs have become the new standard of care for second-line treatment of patients with advanced NSCLC harboring the KRAS^G12C^ mutation, modifying current treatment algorithms (15).

Although the introduction of the first covalent OFF-state inhibitors (sotorasib, adagrasib) has significantly transformed the treatment of NSCLC with the KRAS^G12C^ mutation, their efficacy is limited by toxicity and the rapid development of resistance. The clinical situation is further complicated by the fact that this mutation often co-occurs with STK11/KEAP1 alterations (a negative prognostic factor that shortens survival) and is associated with frequent brain metastases, for which current therapies are suboptimal. In response to these challenges, the medical community is currently developing novel ON (GTP-bound) inhibitors, pan-RAS inhibitors, and combination strategies [combining KRAS inhibitors with immunotherapy, Src homology region 2-containing protein tyrosine phosphatase 2 (SHP2) inhibitors, or Son of Sevenless homolog 1 (SOS1)]. The need to optimize these regimens and balance their toxicity represents an important area of ongoing therapeutic development (16).

The aim of this paper is to present the current state of knowledge regarding sotorasib and adagrasib and to evaluate their potential role in the treatment of NSCLC, with particular emphasis on patients with intracranial disease. Given the limitations of current therapeutic approaches and the rapid development of resistance, there is a constant need to seek more effective strategies, which is generating growing interest in the scientific community in these new molecules. An analysis of available clinical trials regarding the efficacy and safety profiles of these drugs may contribute to a better understanding of their significance in modern lung cancer therapy and identify directions for further research, including the development of ON state inhibitors and combination therapies.

## 2 Materials and methods

### 2.1 Study designs

The systematic review was conducted in accordance with the Preferred Reporting Items for Systematic Reviews and Meta-Analyses (PRISMA) 2020 guidelines. Although the final synthesis is focused on a limited number of Phase III trials, a rigorous systematic approach was prospectively chosen to eliminate selection bias, objectively filter out confounding early-phase or observational data, and definitively confirm the current landscape of Level 1 evidence regarding these targeted therapies. The primary aim was to comprehensively assess the clinical efficacy and safety of sotorasib and adagrasib in NSCLC, particularly emphasizing their comparative efficacy and safety profiles against standard docetaxel chemotherapy.

The analysis included phase III Randomized Controlled Trial (RCTs) evaluating sotorasib or adagrasib in adult patients with KRAS^G12C^-mutated advanced NSCLC, including long-term follow-up reports, predefined subgroup analyses, and patient-reported outcome (PROs) analyses derived from the same parent trial programs.

The review protocol was registered in the International Prospective Register of Systematic Reviews (PROSPERO) under registration number CRD420261427962.

### 2.2 Eligibility criteria and PICO framework

The research focused on evaluating the efficacy and safety of sotorasib and adagrasib therapies. The PICO model was used to structure and focus the literature review.

- Population: The target population consists of adult patients diagnosed with advanced or metastatic *KRAS*^G12C^-mutated NSCLC, regardless of gender.

- Intervention: Interventions included sotorasib or adagrasib administered as targeted monotherapy.

- Comparison: Comparators included standard second-line chemotherapy, specifically docetaxel.

- Outcome: The primary and secondary outcomes focused on clinical efficacy, safety, and health-related quality of life. Efficacy endpoints encompassed objective response rate (ORR), disease control rate (DCR), progression-free survival (PFS), overall survival (OS), duration of response (DoR), complete response (CR), partial response (PR), stable disease (SD), and progressive disease (PD). To address central nervous system (CNS) efficacy, intracranial ORR (iORR), intracranial DCR (iDCR), central nervous system progression-free survival (CNS PFS), and CNS time-to-progression (CNS TTP) were systematically evaluated. Safety was assessed based on the incidence and severity of treatment-related adverse events (TRAEs) according to Common Terminology Criteria for Adverse Events (CTCAE). Additionally, PROs and quality of life (QOL) metrics.

Studies published in English between 2023 and 2026 were eligible for inclusion. The review considered prospective RCTs (phase III) evaluating sotorasib or adagrasib as monotherapy against standard chemotherapy in adult patients with advanced *KRAS*^G12C^-mutated NSCLC. This strict restriction to Phase III trials was applied intentionally to filter out the potential bias of single-arm Phase II studies (such as CodeBreaK 100 and KRYSTAL-1) and provide a synthesis grounded exclusively in Level 1 evidence.

In addition to primary trial reports, secondary publications derived from the same parent clinical trial programs—including predefined subgroup analyses, PROs, and extended follow-up reports—were also included when they provided additional clinically relevant efficacy or safety data. Multiple publications originating from the same trial were treated as linked records and synthesized under a single trial program.

Exclusion criteria comprised review articles, preclinical studies (animal or cellular models), conference abstracts or posters without complete data publications lacking relevant efficacy or safety outcomes related to sotorasib or adagrasib therapy, and randomized trials assessing alternative dosing regimens that lacked a standard chemotherapy control arm.

A summary of the inclusion and exclusion criteria based on the PICO framework is presented in **Table 1**.

**Table 1:** Eligibility criteria based on the PICO framework.

Component	Criteria
Population (P)	Adult patients diagnosed with advanced or metastatic *KRAS^G12^*^C^-mutated NSCLC, regardless of gender.
Intervention (I)	Sotorasib or adagrasib administered as targeted monotherapy.
Comparison (C)	Standard second-line chemotherapy, specifically docetaxel monotherapy.
Outcomes (O)	Systemic Efficacy: ORR, DCR, PFS, OS, DoR, CR, PR, SD, PD CNS Efficacy: iORR, iDCR, CNS PFS, CNS TTP Safety: TRAEs (incidence and severity graded by CTCAE criteria) PROs/Quality of Life: FACT-G, PRO-CTCAE, EQ-5D VAS, EORTC QLQ-C30, and EORTC QLQ-LC13 scores
Study design	Prospective, phase III RCTs. Linked secondary publications from the same parent trial programs (predefined subgroup analyses, PROs/QOL reports, and extended long-term follow-up analyses) were also included.
Publication years	2023–2026
Language	English
General exclusion criteria	Review articles, preclinical studies (*in vitro*, cellular, or animal models), conference abstracts or posters without complete data, publications lacking relevant efficacy/safety outcomes, and randomized trials assessing alternative dosing regimens without a standard chemotherapy control arm.

### 2.3 Search and selection process

A systematic search of PubMed, Scopus, and ClinicalTrials.gov was performed to identify relevant studies published between January 1, 2023, and May 31, 2026. The final search was conducted on June 15, 2026.

The following keywords were used with database-specific adaptations: (“sotorasib” OR “adagrasib”) AND (“non-small cell lung cancer” OR “NSCLC”) AND (“KRAS^G12C^” OR “KRAS mutation”).

To ensure completeness, a manual search of the reference lists of all eligible studies was also performed. Two researchers (JuP. and NP.) independently screened titles, abstracts, and full-text articles using predefined eligibility criteria. Any discrepancies were resolved through discussion with two additional reviewers (JaP. and NG.).

ClinicalTrials.gov records were cross-checked against peer-reviewed publications to avoid duplicate inclusion of registry entries and published reports. When multiple publications originated from the same parent clinical trial, these were treated as linked records and synthesized under a single trial program.

**Figure 1:**
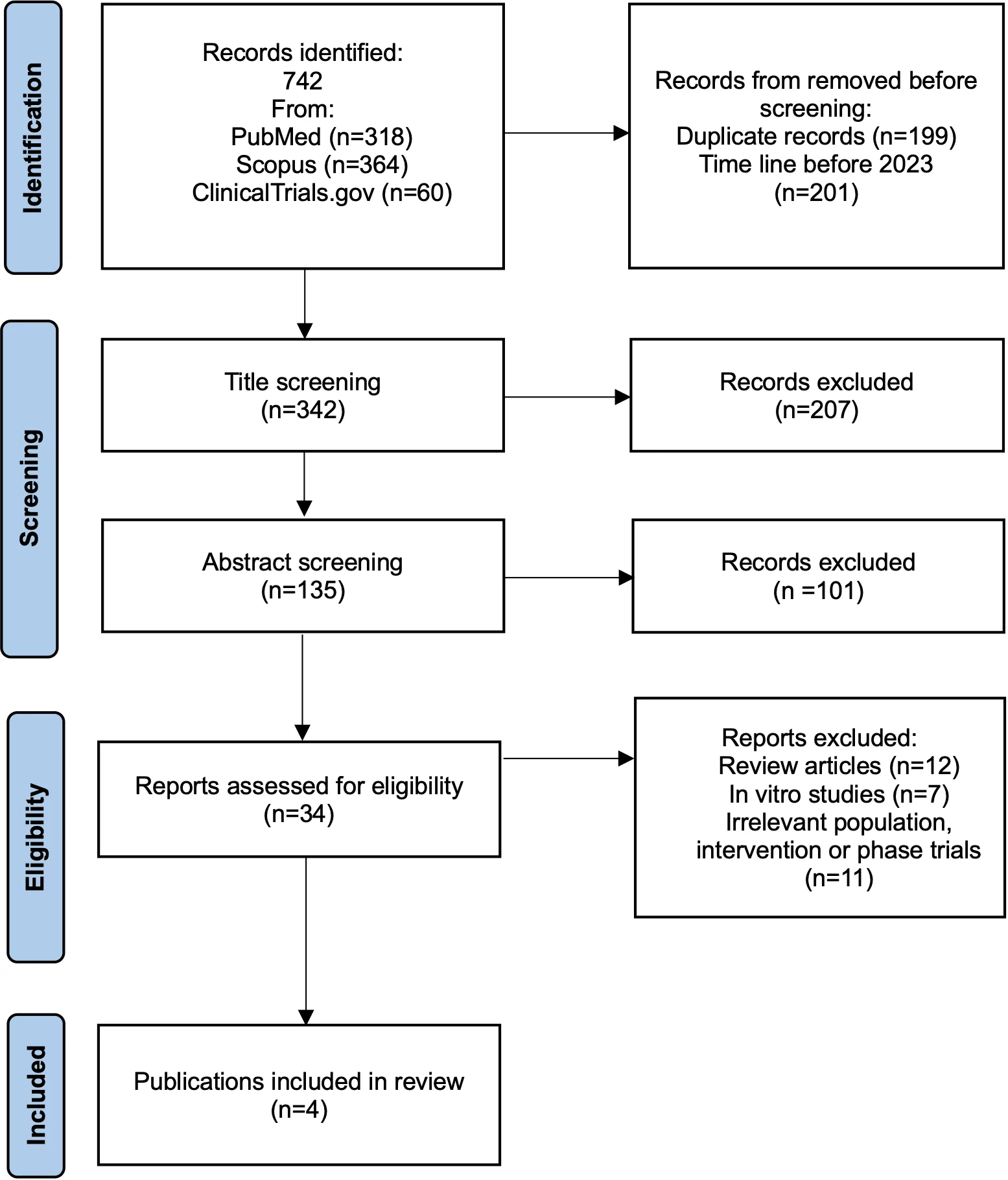
Preferred reporting items for systematic reviews and meta-analyses (PRISMA) flow diagram of study identification, inclusion, and exclusion.

The database search identified 742 records. Before screening, 400 records were removed due to duplication (n=199) or publication timeline restrictions (n=201). A total of 342 records underwent title screening, with 207 being excluded. Subsequently, 135 records were screened by abstract, leading to the exclusion of 101 records. The remaining 34 reports were assessed for full-text eligibility, of which 30 were excluded based on predefined criteria. Ultimately, four eligible publications representing two unique clinical trial programs were included in the final qualitative synthesis. The study selection process is presented in the PRISMA flow diagram in **Figure 1**.

### 2.4 Data extraction

The review included four publications derived from two major clinical trial programs (CodeBreaK200 and KRYSTAL-12). These publications collectively reported data from 798 unique patients, with linked secondary reports providing additional subgroup, long-term follow-up, and PROs data.

Data extraction was performed independently by two reviewers using a standardized form, with any discrepancies resolved through discussion or consultation with a third reviewer. Extracted variables included parent trial program, publication type (primary efficacy, subgroup, follow-up, or PRO report), study population, treatment regimen, and efficacy and safety outcomes. Specifically, extracted data encompassed study characteristics (author, year, study design), patient demographics (total number of patients, age, gender), intervention details (dosage, regimen, monotherapy vs. combination therapy), and outcome measures related to efficacy (ORR, PFS, OS, DoR, CR, PR, SD, PD) and safety (incidence and severity of TRAEs).

Due to the limited number of unique Phase III randomized controlled trials (k=2) and critical methodological divergence between the trial protocols, a formal quantitative meta-analysis was not performed. Specifically, the high and asymmetrical crossover rate in the CodeBreaK 200 trial (where 26% of patients in the control arm crossed over to sotorasib), alongside distinct differences in pharmacokinetic profiles, and varying central nervous system (CNS) baseline imaging mandates and tracking intervals, introduced significant clinical heterogeneity. Pooling these data quantitatively could yield mathematically misleading pooled effect sizes, particularly for OS. Therefore, the data were synthesized qualitatively to preserve the integrity of individual trial outcomes.

### 2.5 Assessment of risk of bias in the included studies

**Figure 2:**
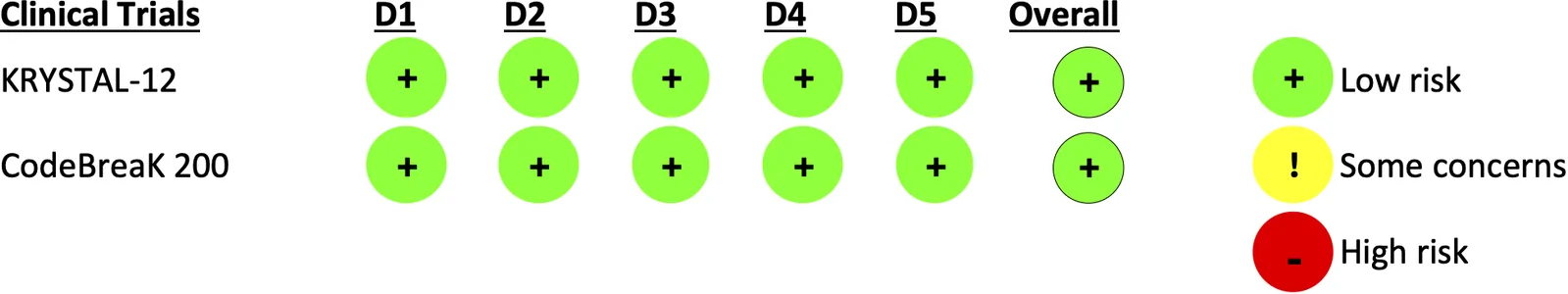
Risk of bias assessment for the included RCT (KRYSTAL-12 and CodeBreaK200) using the Cochrane Risk of Bias 2.0 (RoB 2.0) tool. Risk of bias is evaluated across five specific domains: D1: Bias arising from the randomisation process; D2: Bias due to deviations from the intended interventions; D3: Bias due to missing outcome data; D4: Bias in measurement of the outcome; and D5: Bias in selection of the reported result. The green circles with a plus sign (+) indicate a low risk of bias.

Risk of bias was assessed at the level of the parent randomized trials using the updated Cochrane Risk of Bias tool for randomized trials (RoB 2.0) (43). This tool evaluates five domains: (1) bias arising from the randomization process, (2) bias due to deviations from intended interventions, (3) bias related to missing outcome data, (4) bias in outcome measurement, and (5) bias in the selection of reported results. Two independent reviewers applied RoB 2.0 to all parent trials, recording supporting evidence and providing judgments of low risk, high risk, or some concerns for each domain. Discrepancies were resolved through discussion or adjudication by a third reviewer. For linked secondary publications (such as subgroup, PRO, and long-term follow-up reports), risk-of-bias judgments were anchored to the corresponding parent trial, with additional consideration of reporting completeness and outcome-specific limitations. The domain-level RoB 2.0 summary for all parent randomized studies included in the review is shown in **Figure 2**.

## 3 Pharmacological profiles: sotorasib and adagrasib

**Figure 3:**
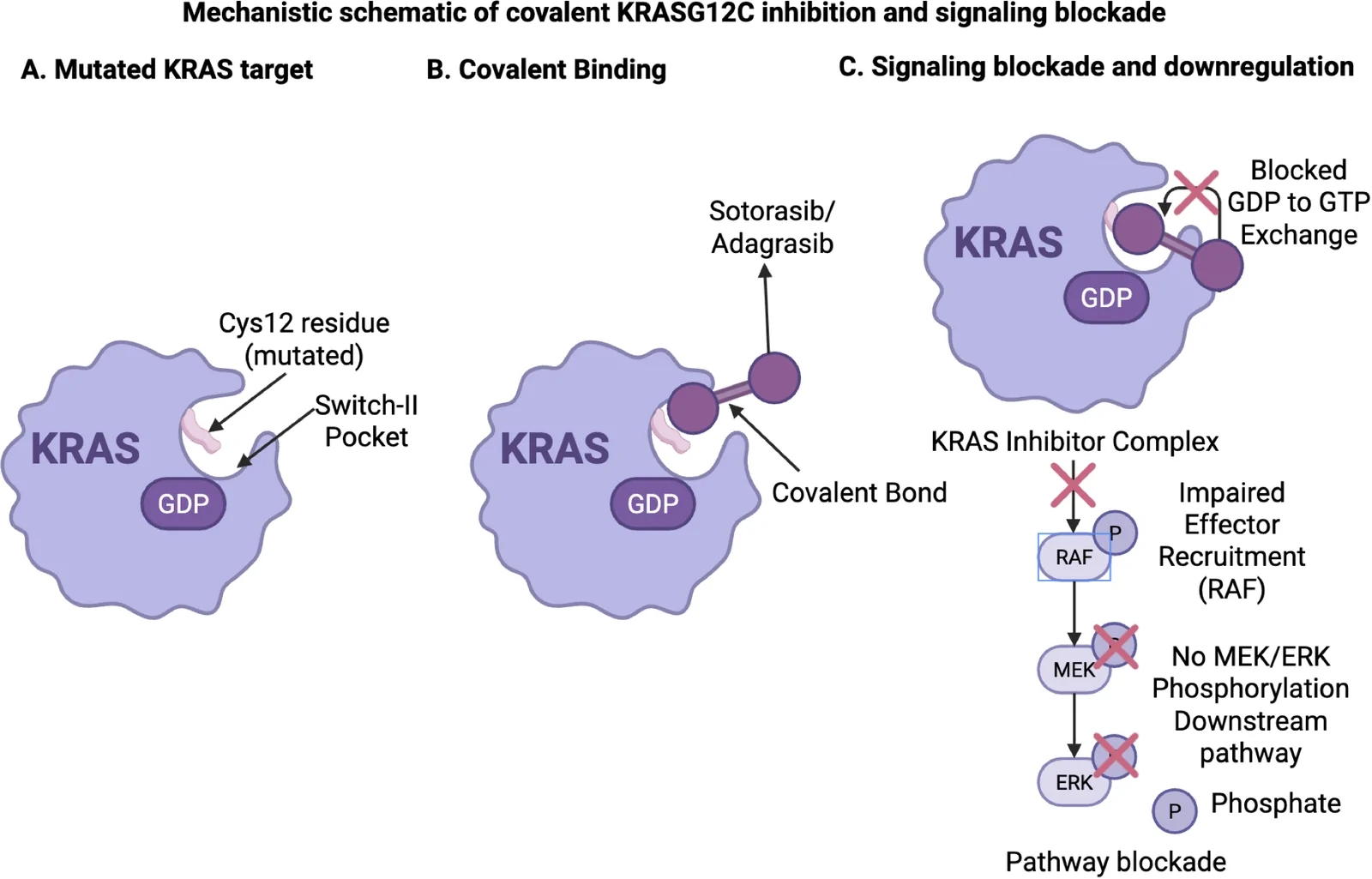
Mechanism of covalent KRASG12C inhibition and downstream pathway blockade. **(A)** Sotorasib/adagrasib targeted allosteric Switch-II pocket adjacent to the mutated Cys12 residue while KRAS is bound to GDP. **(B)** Direct inhibitors form an irreversible, covalent bond with the mutant cysteine. **(C)** The resulting complex permanently locks KRAS in its inactive "OFF" state, preventing GDP-to-GTP exchange. This blocks the recruitment of RAF and eliminates downstream phosphorylation of MEK and ERK, leading to tumor growth arrest.

The KRAS^G12C^ mutation is uniquely notable for its propensity to induce a hyperexcitable state within the protein, rather than a constitutively active one. Both sotorasib and adagrasib represent a novel class of mutant-specific small-molecule inhibitors engineered specifically to exploit this dynamic. Mechanistically, these agents achieve targeted pathway suppression by antagonizing the inactive, GDP-bound conformation of KRAS^G12C^. They accomplish this through highly selective, covalent binding to the mutant cysteine (Cys12) residue located within the switch-II pocket of the KRAS protein. By permanently locking KRAS^G12C^ in this inactive state, the inhibitors sterically block nucleotide exchange, effectively uncoupling the mutant KRAS from its downstream signaling cascades. This results in the rapid extinguishment of the MAPK/ERK pathway, manifesting as a complete abrogation of the phosphorylation of key downstream effectors—specifically MEK and ERK—ultimately arresting tumor cell proliferation (17). The detailed schematic representation of this covalent inhibition mechanism and its impact on downstream signaling cascades is illustrated in **Figure 3**.

The clinical translation of this molecular mechanism led to rapid global regulatory milestones. Initial therapeutic advances were driven by early-phase clinical programs evaluating monotherapy performance. For sotorasib (AMG 510), clinically meaningful objective responses demonstrated in the single-arm Phase I/II CodeBreaK 100 trial prompted the US Food and Drug Administration (FDA) to grant accelerated approval in May 2021, establishing it as the first-ever approved targeted therapy against KRAS (18, 19). This was followed by the European Medicines Agency’s (EMA) conditional marketing authorization in January 2022 (20).

Similarly, for adagrasib (MRTX849), pivotal efficacy data from the Phase I/II KRYSTAL-1 cohort led to FDA accelerated approval in December 2022. The EMA subsequently granted conditional marketing authorization for adagrasib in January 2024. While these early-phase single-arm studies laid the foundational framework for KRAS^G12C^ inhibition, confirmatory randomized Level 1 evidence was subsequently provided by the Phase III trials analyzed in this systematic review—namely CodeBreaK 200 for sotorasib and KRYSTAL-12 for adagrasib—which directly evaluated these targeted agents against standard docetaxel chemotherapy (21, 22).

While sharing an identical mechanism of action, sotorasib and adagrasib possess distinctly divergent pharmacokinetic (PK) and pharmacodynamic (PD) profiles, which also correspond to different tolerability profiles in clinical settings. A comprehensive comparison of the key pharmacological parameters for both inhibitors is summarized in **Table 2**. Sotorasib is administered at a standard dose of 960 mg orally once daily (23). It exhibits a relatively short plasma half-life of approximately 5.5 hours. Conversely, adagrasib is administered at a standard dose of 600 mg orally twice daily and is characterized by a significantly prolonged half-life of approximately 23 hours (24). A hallmark of adagrasib’s pharmacodynamics is its slow target dissociation rate; this property supports prolonged target coverage and sustained inhibition of the MAPK/ERK pathway throughout the entire dosing interval (25).

**Table 2:** Key pharmacokinetic and pharmacodynamic profiles of sotorasib and adagrasib.

Pharmacological parameter	Sotorasib (AMG 510)	Adagrasib (MRTX849)
Standard dosing	960 mg PO once daily	600 mg PO twice daily
Elimination half-life (t_1/2_​)	~ 5.5 hours	~ 23 hours
Primary metabolism	CYP3A4 (major)	CYP3A4 (major)
Cellular affinity (IC_50_)	~ 90 nM	~ 14 nM
CNS penetration	Clinically limited	High (therapeutic CSF levels)

The table highlights critical differences in dosing schedules, elimination half-lives, and CNS penetration capacity. Both agents are primarily metabolized via the cytochrome P450 3A4 (CYP3A4) pathway. PO, per os (orally); IC_50_, half-maximal inhibitory concentration; CSF, cerebrospinal fluid (23, 24).

Furthermore, a critical clinical differentiator between the two compounds is their reported CNS penetration. Adagrasib was deliberately optimized to cross the blood-brain barrier (BBB), successfully achieving therapeutic concentrations in the cerebrospinal fluid (CSF) in preclinical and early clinical evaluations. While sotorasib has also demonstrated notable intracranial efficacy in clinical trials, limited data exist regarding its direct CSF penetration. These foundational pharmacological differences may also contribute to variations in their safety profiles, such as adagrasib being associated with a higher prevalence of gastrointestinal side effects and Grade ≥3 adverse events leading to dose reductions, whereas sotorasib presents a generally more favorable safety profile concerning severe toxicities. However, in the absence of head-to-head randomized trials, any comparative interpretations regarding pharmacokinetics, CNS penetration, and tolerability remain strictly indirect observations (26).

## 4 Results

### 4.1 Randomized phase III CodeBreaK 200 trials (NCT04303780)

A total of 345 patients with advanced NSCLC harboring the KRAS^G12C^ mutation participated in an open-label, multicenter, Phase III RCTs evaluating the efficacy, safety, and tolerability of sotorasib. Enrolled patients were required to have experienced disease progression following prior platinum-based chemotherapy and immunotherapy with PD-1 and PD-L1 inhibitors. Participants were randomly assigned in a 1:1 ratio to two groups. The treatment group (n=171) received sotorasib at a dose of 960 mg once daily, while the control group (n=174) received docetaxel 75 mg/m² intravenously every 3 weeks.

With a median follow-up time of 17.7 months, the median duration of exposure to sotorasib was 19.9 weeks, while that for docetaxel was 12 weeks. At the time of analysis, 22 patients (13%) in the treatment group and 7 (4%) in the control group were still receiving treatment. A key feature of the protocol was the permitted crossover—46 patients (26%) from the docetaxel group switched to sotorasib after confirmed disease progression.

The primary endpoint was PFS, which, according to the Blinded Independent Central Review (BICR), was 5.6 months in the study group and 4.5 months in the control group (p=0.0017), with an overall hazard ratio (HR) for progression or death of 0.66 (95% CI: 0.51–0.86). Landmark analysis further revealed that the 6-month PFS rate was approximately 43.0% in the sotorasib arm versus 36.0% in the docetaxel arm. Subsequently, the 12-month PFS rate was significantly higher in the sotorasib arm than in the docetaxel arm, at 24.8% versus 10.1%, demonstrating a more than two-fold increase in sustained, long-term disease control at one year.

For secondary endpoints, the ORR in the targeted therapy group was 28.1%, compared with 13.2% in the chemotherapy arm (p<0.0001). Similarly, the DCR was 82.5% versus 60.8%. The median DoR in patients receiving sotorasib was 8.6 months versus 6.8 months in the control group. In contrast, the median OS was higher in patients receiving docetaxel (11.3 months) than in those receiving sotorasib (10.6 months). However, this survival analysis was heavily confounded by a high rate of patient crossover; as noted in the protocol, 46 patients (26%) from the docetaxel control arm switched to receive sotorasib after disease progression. Consequently, when accounting for this extensive crossover and subsequent post-progression therapies, overall survival does not accurately reflect the comparative efficacy of the primary treatment, rendering PFS and ORR more robust indicators of the clinical benefit observed in this trial. Furthermore, 36% of patients received subsequent therapy after treatment with sotorasib, compared with 42% after docetaxel. The study also demonstrated benefits in the subpopulation of patients with CNS metastases. In an exploratory analysis, the median time to progression of intracranial lesions was longer in the sotorasib group compared to the docetaxel group, at 15.8 months versus 10.5 months (HR 0.52, 95% CI 0.26–1.0).

The safety profile of sotorasib was consistent with previously reported data, and the incidence of TRAEs of any grade was lower in the study group (70%) compared with the control arm (86%). Grade ≥3 TRAEs were reported in 33% of patients receiving sotorasib and in 40% of those treated with docetaxel.

In the sotorasib group, the most common TRAEs of any grade were diarrhea (34%), nausea (14%), elevated alanine aminotransferase (ALT) levels (10%), and elevated aspartate aminotransferase (AST) levels (10%); these events were generally mild or moderate in severity. Among adverse events of grade ≥3 following sotorasib administration, the most commonly observed were diarrhea (12%), elevated ALT (8%), and elevated AST (5%). It is worth noting that all of these Grade ≥3 hepatic and gastrointestinal events resolved following the implementation of standard dose-modification strategies, such as temporary treatment interruption or dose reduction.

By comparison, docetaxel’s toxicity profile was different and dominated by hematologic and systemic complications, with the most common events of any grade including fatigue (25%), alopecia (21%), nausea (20%), and diarrhea (19%). Grade ≥3 events in the control group mainly involved neutropenia (12%), asthenia (3%), and anemia (3%). Permanent discontinuation of therapy due to toxicity was similar in both groups, at 10% for sotorasib and 11% for docetaxel. Toxicity-related fatalities were reported in one patient in the sotorasib group (interstitial lung disease) and in two patients in the docetaxel group (intestinal obstruction and multiple organ failure) (27).

#### 4.1.1 CNS efficacy: *post hoc* analysis of the CodeBreaK 200

The results of the CodeBreaK 200 study are supplemented by a detailed *post hoc* analysis aimed at evaluating the drug’s activity in the CNS, conducted with a median follow-up of 20.0 months for this subgroup of patients. Baseline CNS lesions were identified in 40 patients (23%) in the sotorasib arm and in 29 (17%) in the docetaxel group. In the full CNS analysis population, the median CNS PFS was significantly longer with sotorasib, at 9.6 months compared with 4.5 months for docetaxel (HR = 0.43; 95% CI, 0.20–0.92), and the median CNS TTP was significantly prolonged, reaching 11.6 months versus 6.0 months in the chemotherapy arm (HR = 0.63; 95% CI, 0.25–1.62). The median OS in the analyzed subgroup was similar in both arms, at 11.7 and 11.9 months, respectively (HR = 1.4; 95% CI, 0.71–2.81).

In the subpopulation of patients with at least one measurable lesion in the CNS (≥10 mm), assessed according to the modified Response Assessment in Neuro-Oncology Brain Metastases (RANO-BM) criteria, the iORR (defined as a reduction in CNS tumor size of ≥30%) was twice as high for sotorasib as for docetaxel (33.3% vs. 15.4%). A high concordance was also observed between systemic and iDCR, which was 88% in the treatment arm versus 54% in the control arm, accompanied by a longer duration of treatment with sotorasib (median treatment duration of 6.8 vs. 3.0 months). The incidence of TRAEs in patients with baseline CNS lesions was consistent with the overall population of the CodeBreaK 200 study, and the proportion of Grade ≥3 events was similar in both groups (30% vs. 28%). The most common Grade ≥3 TRAEs for sotorasib were diarrhea (10%) and elevated ALT (7.5%), while for docetaxel they were neutropenia (10%) and fatigue (6.9%). Toxicity led to treatment discontinuation in one patient receiving sotorasib; in the docetaxel arm, no discontinuations were reported for this reason, although one treatment-related death due to intestinal obstruction was recorded (28).

#### 4.1.2 Patient-reported outcomes in the CodeBreaK 200 trial

The analysis PROs results and a detailed QOL assessment serve as an important complement to the clinical evaluation of the CodeBreaK 200 study. A dedicated questionnaire analysis demonstrated that patients receiving sotorasib experienced significantly fewer treatment-related side effects than those in the docetaxel arm. In the Functional Assessment of Cancer Therapy - General (FACT-G) questionnaire, the odds ratio (OR) for selecting a more favorable response category in favor of sotorasib was 5.7 (p<0.001) at week 12, and the percentage of patients reporting treatment-related burden remained stable over time, whereas it increased significantly in the chemotherapy group. An analysis using the Patient-Reported Outcomes Common Terminology Criteria for Adverse Events (PRO-CTCAE) confirmed better tolerability of sotorasib, which at week 12 was associated with less severe pain (OR 2.9), muscle pain (OR 4.4), joint pain (OR 4.2), and mouth or throat sores (OR 4.3), although the incidence of these symptoms remained similar in both groups. Furthermore, these symptoms impaired the patients’ daily functioning to a much lesser extent in the study arm, which was particularly evident with regard to joint pain (OR 10.7), muscle pain (OR 3.9), and pain (OR 3.2). In terms of overall QOL as assessed by the EuroQol 5-Dimension Visual Analog Scale (EQ-5D VAS), parameters stabilized in the sotorasib group, whereas in the docetaxel group there was a marked deterioration in quality of life both at an early stage (change of 1.5 vs. –8.4 on day 5 of the cycle) and at week 12 (2.2 vs. –5.8). Patients taking the KRAS^G12C^ inhibitor also reported a significant improvement in the domains of mobility (OR 2.8), self-care (OR 2.9), and performing routine activities (OR 1.9) compared to baseline. Among the 38 scales and symptoms assessed using the European Organization for Research and Treatment of Cancer Quality of Life Questionnaire Core 30 (EORTC QLQ-C30) and the European Organization for Research and Treatment of Cancer Quality of Life Questionnaire Lung Cancer 13 (EORTC QLQ-LC13), as many as 36 score indicators favored sotorasib, 23 of which reached statistical significance (including delayed time to deterioration in overall health, physical functioning, dyspnea, and cough).

Docetaxel proved to be more effective only with regard to vomiting and diarrhea, of which only diarrhea showed a statistically significant difference, which is consistent with the known safety profile of sotorasib. The positive effect of sotorasib on reducing the burden of cough, chest pain, and shortness of breath was also confirmed by the results of the Patient Global Impression of Change (PGIC) and Patient Global Impression of Severity (PGIS) scales, which indicate that this therapy allows for an optimal tolerability profile while maintaining a high quality of life for patients (29).

### 4.2 Randomized phase III KRYSTAL-12 trial (NCT04685135)

This open-label, multicenter, phase III RCTs enrolled 453 patients with locally advanced or metastatic NSCLC who had previously received platinum-based chemotherapy and immunotherapy. All patients were found to have a KRAS^G12C^ mutation. Participants were randomized in a 2:1 ratio. The treatment group (n=301) received 600 mg of adagrasib twice daily by mouth in continuous 3-week cycles, while the control group (n=152) received 75 mg/m² of docetaxel intravenously every 3 weeks.

The primary endpoint of the study, after a median follow-up of 7.2 months, was PFS as assessed by BICR, which was 5.5 months in the treatment group compared with 3.8 months in the control group (p<0.0001); the corresponding hazard ratio (HR) for disease progression or death was 0.58 (95% CI: 0.45–0.76). Crucially, exploratory landmark evaluation revealed a statistically higher 6-month PFS rate of 44.6% for the adagrasib cohort compared to 30.1% for docetaxel. At the 12-month milestone, despite progressive sample size attrition with only 19 patients remaining at risk, adagrasib maintained an estimated PFS rate of approximately 22.0% versus less than 8.0% for the chemotherapy control. These results were consistent with the investigator’s assessment, in which the median PFS was 5.4 months and 2.9 months, respectively.

Among the study’s secondary endpoints, the ORR in the adagrasib group was 32%, comprising a CR in 1% of patients and a PR in 31% of participants, whereas in the control arm, the ORR was only 9% (of which 0% were complete responses). The DCR reached 78% in the treatment arm compared with 59% in the control arm; when combined with a median DoR of 8.3 months for adagrasib and 5.4 months for docetaxel, this confirms a significant advantage in the antitumor effect of targeted therapy. Notably, among patients who achieved an objective response, 64.0% in the adagrasib group maintained an ongoing, uninterrupted response at 6 months, compared to only 39.0% in the docetaxel arm, underlining the greater stability of target-specific inhibition over conventional cytotoxic treatment. The median OS for patients receiving adagrasib was 14.1 months, compared to 10.3 months in the docetaxel group.

A key aspect of the study was the evaluation of adagrasib’s efficacy in patients with CNS metastases. In the population of patients with baseline brain metastases, assessed according to the CNS-adapted Response Evaluation Criteria in Solid Tumors (RECIST v1.1), the iORR was 24% in the adagrasib group compared with 11% in the docetaxel group. It is worth noting that complete intracranial responses were observed in 14% of patients receiving adagrasib and in 8% of those treated with docetaxel. The iDCR reached 82% in the adagrasib group and 56% in the docetaxel group. Furthermore, in the subpopulation of patients with at least one measurable lesion in the CNS, the rate of objective intracranial responses was significantly higher for adagrasib, at 40%, compared with 11% for docetaxel, confirming the drug’s high activity within the CNS.

An analysis of the safety profile showed that TRAEs of any severity occurred in 94% of patients in the adagrasib group and in 86% in the docetaxel group. Grade 3 or higher events were reported in 47% of patients treated with adagrasib and in 46% treated with docetaxel. The most common events in the study group were gastrointestinal symptoms, including diarrhea (53%), vomiting (35%), and nausea (34%), while in the control group, diarrhea (31%), anemia (30%), and asthenia (27%) were the most common. Despite more frequent dose modifications in the adagrasib group (dose reduction in 48% of patients), the percentage of permanent treatment discontinuations due to toxicity was lower, at 8%, compared to 14% in the docetaxel group (30).

### 4.3 Summary of clinical trials

A synthesis of the results from Phase III registration trials (CodeBreaK 200 and KRYSTAL-12) indicates that both targeted therapies represent a clinically relevant alternative for advanced NSCLC with the KRAS^G12C^ mutation compared with the reference arm using docetaxel. Both sotorasib and adagrasib achieve highly comparable and reproducible systemic efficacy as measured by PFS. Across the respective Phase III trials, adagrasib was associated with a numerically higher ORR and longer median OS, although these cross-trial observations should be interpreted cautiously because no head-to-head randomized comparison is available. This compound is characterized by a higher incidence of TRAEs, including Grade 3–4 events, which are dominated by bothersome gastrointestinal symptoms and transient elevations in liver enzymes. Sotorasib, on the other hand, offers a more favorable safety profile, which directly translates into favorable HRQoL outcomes—patients taking this drug reported significant stabilization of their general condition and considerably less treatment-related burden than with conventional chemotherapy.

It should be noted, however, that a direct comparison in this area is hampered by a significant asymmetry in the data—the sotorasib arm in the CodeBreaK 200 trial has been the subject of dedicated, widely published analyses of structured PROs, whereas analogous, detailed PRO reports for adagrasib in the KRYSTAL-12 trial have not yet been fully published as an independent study, which makes it difficult to conduct an unambiguous, direct comparison of patients’ quality of life between the two drugs.

Both sotorasib and adagrasib have demonstrated intracranial activity in clinical studies. However, differences in blood-brain barrier penetration and intracranial efficacy should be interpreted cautiously and cannot be directly compared in the absence of head-to-head randomized trials.

**Table 3** presents a detailed, direct comparison of the drugs’ efficacy parameters and TRAE profiles for both studies discussed.

**Table 3:** Summary of clinical trials included in systematic review.

Study	CodeBreaK200	KRYSTAL-12
Population	345 patients	453 patients
Intervention	Sotorasib	Adagrasib
Type of examination	Phase III RCTs	Phase III RCTs
Year of study	2023	2025
Median PFS	5.6 months	5.5 months
6 months PFS	43%	44.6%
12 months PFS	24.8%	22%
ORR	28.1%	32%
CR	2.1%	1%
PR	26%	31%
Median DoR	8.6 months	8.3 months
Median OS	10.6 months	14.1 months
DCR	82.5%	78%
iDCR	88%	82%
Median CNS PFS	9.6 months	NR
iORR	33.3%	24%
Median CNS TTP	11.6 months	NR
TRAEs Any Grade	70%	94%
TRAEs Grade ≥3	33%	47%
Qol/PROs assessment	Significant improvement vs docetaxel	Data currently emerging
Citation	(27–29)	(30)

Not reported (NR).

## 5 Discussion

This review summarizes current evidence regarding KRASG12C-targeted therapies in thoracic oncology, marking the transition from therapeutic stagnation to the era of precision medicine targeting KRAS^G12C^ mutations. For decades, this oncogene was considered “undruggable” due to the lack of deep allosteric pockets. The overcoming of this barrier by covalent inhibitors that bind to the Switch-II pocket in its inactive GDP-bound configuration has transformed treatment algorithms. Data from Phase III trials—CodeBreaK 200 and KRYSTAL-12—demonstrate that sotorasib and adagrasib represent clinically active and generally manageable alternatives to docetaxel-based chemotherapy. However, optimizing these results requires an analysis of pharmacokinetic differences, biological mechanisms of resistance, and upcoming therapeutic innovations.

The indirect synthesis of efficacy data from the CodeBreaK 200 and KRYSTAL-12 trials supports the clinical benefit, demonstrating the efficacy of targeted KRAS G12C inhibition over standard docetaxel chemotherapy. Across both independent cohorts, cytotoxic chemotherapy consistently underperformed, yielding limited median PFS values (3.8 and 4.5 months) and severely restricted response durations. By comparison, sotorasib and adagrasib demonstrated broadly comparable efficacy, with nearly identical median PFS values of 5.6 and 5.5 months, respectively. However, median PFS alone does not fully capture the differences observed between the two studies. Landmark survival analyses and response kinetics provide additional context for interpreting the temporal patterns of clinical benefit, although these observations should be interpreted cautiously given the absence of head-to-head randomized comparisons.

In the CodeBreaK 200 trial, sotorasib demonstrated durable progression-free survival during longer follow-up. With a median follow-up of 17.7 months, the PFS curve showed a plateau at later time points, with a 12-month PFS rate of 24.8% and a hazard ratio (HR) for disease progression or death of 0.66 (95% CI: 0.51–0.86). In the KRYSTAL-12 trial, adagrasib demonstrated favorable early PFS outcomes, with a 6-month PFS rate of 44.6% compared with 30.1% for docetaxel and a hazard ratio of 0.58 (95% CI: 0.45–0.76). However, these observations arise from separate clinical trials with different follow-up durations and should therefore be interpreted cautiously rather than as direct evidence of differences between the two agents.

From a strict pharmacological and pharmacokinetic standpoint, this rapid initial divergence and high early disease control achieved by adagrasib has been hypothesized to relate to its distinct molecular dynamics. Adagrasib possesses a prolonged systemic half-life of approximately 23 hours and utilizes a high-dose, twice-daily (600 mg BID) regimen. This specific configuration may support continuous target coverage, which could contribute to adagrasib’s high localized efficacy, driving a 32.0% ORR and an ongoing, sustained tumor response in 64.0% of responding individuals at the 6-month milestone. Nevertheless, establishing a definitive causal relationship between these pharmacokinetic properties and clinical efficacy requires head-to-head randomized trials; currently, such comparative mechanisms must be regarded purely as indirect observations.

Despite these compelling dynamics, the extreme end of the adagrasib survival tail remains highly immature due to the trial’s short 7.2-month median follow-up, characterized by rapid sample attrition with a mere 19 patients remaining under observation at one year. Consequently, while the therapeutic benefit of both targeted agents over docetaxel is well-documented, any direct graphical superimposition or formal visual overlay of the CodeBreaK 200 and KRYSTAL-12 survival curves would be methodologically inappropriate, scientifically flawed, and highly vulnerable to cross-trial comparison bias. Long-term survivorship comparisons can only be definitively established once the adagrasib data reaches full clinical maturity.

Beyond progression-free intervals, the raw median Overall Survival (OS) differences highlighted across these datasets—specifically the numerical variance between adagrasib (14.1 months) and sotorasib (10.6 months)—must be critically interpreted. In the CodeBreaK 200 trial, sotorasib’s median OS was heavily confounded and artificially suppressed by a massive 26% patient crossover rate, wherein 46 patients initially assigned to the docetaxel control arm switched to sotorasib upon disease progression. This ethical crossover protocol inherently dilutes the true survival delta between the study groups by falsely elevating the survival outcomes of the chemotherapy control arm. Conversely, the final OS trajectories remain highly multifactorial; elements such as unequal lines of subsequent post-progression therapies, distinct institutional follow-up patterns, and patient baseline characteristics across the two unlinked trial protocols heavily distort any crude cross-trial OS comparison. Therefore, the numerical gap in median OS does not reflect a true therapeutic inferiority of sotorasib, but rather underscores the profound confounding impact of protocol-permitted treatment crossover and heterogeneous subsequent care patterns.

However, this observation is multifactorial; other elements, such as the influence of diverse subsequent therapies, the limitations of statistical power inherent in the study design, the duration of follow-up, and the varying nature of post-progression treatments, also likely contributed to the final survival outcomes. The numerically higher OS observed with adagrasib may be associated with its pharmacokinetic profile, such as a long half-life (approximately 23 hours) and twice-daily dosing, which could promote more continuous suppression of the MAPK pathway. However, across independent trials with different patient cohorts and follow-up durations, such causal interpretations remain. When interpreting survival data across KRAS G12C inhibitor trials, the confounding impact of ethical crossover protocols must be critically considered. For instance, the lack of an OS advantage for sotorasib in the CodeBreaK 200 trial is widely acknowledged to be an artifact of extensive post-progression crossover from the chemotherapy arm to targeted treatment. Consequently, evaluating the true survival benefit of targeted therapy over chemotherapy purely through intent-to-treat OS analysis is challenging, and efficacy should be assessed through a composite of PFS, duration of response, and clinical benefit (31).

An important clinical consideration that determines the choice of the optimal inhibitor in everyday oncology practice is the ability to cross the blood-brain barrier and elicit an intracranial response. In this regard, both molecules have overcome the pharmacokinetic limitations of older anticancer drugs. A *post hoc* analysis of the CodeBreaK 200 study showed that sotorasib extends the median CNS PFS to 9.6 months and the time to progression in the CNS to 11.6 months. Adagrasib, in turn, demonstrates clinically significant efficacy, achieving an iORR of 40% and intracranial complete responses (iCR) in 14% of patients. However, any definitive comparison of intracranial efficacy between sotorasib and adagrasib is currently precluded by significant methodological discrepancies, including variations in eligibility criteria, distinct intracranial response assessment protocols (e.g., RANO-BM vs. RECIST 1.1), and heterogeneous patient populations across the analyzed trials. Consequently, while adagrasib’s profile appears promising in terms of CNS penetration, current data are insufficient to conclude that one agent is more effective than the other, and these observations must be interpreted with caution in the absence of head-to-head randomized trials.

The numerically higher efficacy reported for adagrasib in its respective Phase III trial occurred alongside a higher incidence of treatment-related adverse events, highlighting a potential trade-off between clinical response and treatment tolerability. In the KRYSTAL-12 study, the incidence of Grade ≥3 TRAEs was 47%, primarily in the form of gastrointestinal symptoms, which necessitated dose reductions in 48% of patients. Sotorasib exhibits a milder safety profile, with a rate of grade ≥3 events at 33% and good control of hepatotoxicity through periodic treatment interruptions. The optimal tolerability of sotorasib is confirmed by PRO analyses, which show a statistically significant improvement on the FACT-G and EORTC QLQ-C30 questionnaires compared to chemotherapy. The available evidence suggests potential differences in efficacy and tolerability profiles; however, treatment selection should be individualized, and direct comparative conclusions cannot be drawn in the absence of head-to-head randomized trials.

Despite the common KRAS^G12C^ mutation, patients’ clinical trajectories vary widely depending on the profile of co-occurring somatic mutations, such as TP53 (40–50%), STK11 (20–30%), and KEAP1 (10–27%). KRAS/TP53 alterations identify immunologically “hot” tumors with high PD-L1 expression, which demonstrate robust responses to immune checkpoint inhibitors (ICI). In contrast, STK11 and KEAP1 mutations induce a “cold” phenotype and treatment resistance through remodeling of the tumor microenvironment (TME) and activation of Nuclear factor erythroid 2-related factor 2 (NRF2). Clinical data confirm that STK11/KEAP1 co-mutations constitute an independent, negative prognostic factor that shortens PFS and OS, making them a key challenge for future targeted strategies. Clinically, these co-alterations are pivotal for treatment selection: STK11/KEAP1-mutated tumors represent a significant unmet need, often necessitating earlier transition to clinical trials or novel combination strategies capable of bypassing NRF2-driven resistance. Conversely, patients with TP53 co-mutations may benefit from earlier integration of immunotherapy given the potential for enhanced ICI response (32, 33). Recent real-world evidence suggests that the KRAS G12C mutation itself may serve as a predictor of improved survival in patients receiving immunotherapy. Such data, when integrated with findings from randomized trials, help to refine our understanding of how KRAS G12C status and its co-occurring mutations influence the efficacy of ICIs (34). Consequently, molecular profiling for these co-mutations is becoming essential to tailor therapeutic sequencing and improve patient outcomes.

The biological heterogeneity of the patient population described above makes it extremely important to compare the results of rigorous RCTs with data from *Real-World Evidence* (RWE). A key study in this regard is the retrospective analysis by Virginia Cancer Specialists, which evaluated the efficacy of sotorasib in second-line and subsequent lines of treatment (2L+) in patients with advanced NSCLC who had previously received immunotherapy and platinum-based therapy. In the 2L population, the median OS for sotorasib was 10.2 months compared with 6.0 months in the docetaxel group, representing a 38% reduction in the risk of death (HR 0.62). These results, free from the crossover bias present in registration trials, provide reliable evidence of a real clinical benefit of KRAS^G12C^ inhibitors in RWE practice (35).

Despite the documented clinical efficacy of first-generation inhibitors, the duration of response to monotherapy is limited by dynamic mechanisms of acquired resistance, which we classify as on-target and off-target changes. On-target resistance results mainly from clonal evolution and the acquisition of secondary mutations within the protein domain, such as Y96C, H95Q/R/W, or R68M. The Y96C mutation, in particular, is critical because it introduces a spatial hindrance in the SWII pocket, completely preventing drug binding, whereas other variants increase the rate of nucleotide exchange, promoting the protein’s transition to an active (GTP-bound) state that is insensitive to current inhibitors. Off-target resistance, on the other hand, involves a biological bypass of the KRAS pathway blockade through the activation of alternative tyrosine kinase receptors (Epidermal Growth Factor Receptor [EGFR], Fibroblast Growth Factor Receptor [FGFR], Human Epidermal Growth Factor Receptor 2 [HER2]) and the accumulation of mutations in other components of the signaling cascade, including BRAF amplification or MAP2K1 activation. The final, clinically extremely challenging issue is phenotypic plasticity, manifested by the transformation of adenocarcinoma into SCLC or the intensification of the epithelial-mesenchymal transition (EMT), which completely eliminates the tumor’s dependence on the original oncogenic driver (36, 37). In this context, serial analysis of circulating tumor DNA (ctDNA) takes on critical importance; thanks to its high sensitivity, it allows for the detection of clonal evolution and the emergence of secondary mutations even before radiological progression, serving as the foundation of modern therapeutic monitoring.

### 5.1 Future perspectives

The dynamic nature of treatment resistance has driven a shift toward combination strategies, such as dual blockade of compensatory feedback loops or vertical inhibition via SHP2 and SOS1. Clinical research is increasingly evaluating these combinations in early-line settings. For adagrasib, the Phase III KRYSTAL-4 study (NCT06875310) is investigating a triple combination with pembrolizumab and chemotherapy as first-line therapy. Furthermore, the KRYSTAL-7 trial (NCT04613596) explores dual immune-targeted blockade (adagrasib plus pembrolizumab) in patients with PD-L1 TPS ≥50%, with preliminary data indicating manageable toxicity profiles (38). Early intervention is also being explored in the neoadjuvant setting, such as in the Phase II Neo-KAN trial (NCT05472623).

Innovative approaches to overcoming resistance include trial NCT07012031, evaluating sotorasib with the antibody-drug conjugate trastuzumab deruxtecan, and studies testing combinations with proteasome inhibitors to induce proteotoxic stress in resistant clones (39).

Next-generation inhibitors, such as divarasib (GDC-6036), represent a significant evolution by exhibiting 5- to 20-fold greater potency and higher selectivity *in vitro* compared to sotorasib and adagrasib. The latest data from the Krascendo program, including the 2026 Krascendo-170 trial, indicate that divarasib combined with pembrolizumab achieved encouraging clinical activity in first-line advanced NSCLC, accompanied by a predictable gastrointestinal tolerability profile that may address the toxicity limitations observed with first-generation inhibitors (26, 40).

Two global Phase III trials, KRASCENDO-1 (NCT06497556) and KRASCENDO-2 (NCT06793215), are currently evaluating divarasib against standard-of-care agents and in first-line combinations, respectively. The results of these studies will clarify its clinical role. Ultimately, the successful application of these targeted strategies depends on the widespread implementation of molecular profiling (NGS) at diagnosis, in alignment with ESMO and NCCN guidelines, to ensure patients are matched to the most appropriate therapeutic pathway (41, 42).

In summary, the current treatment of NSCLC with the KRAS^G12C^ mutation is undergoing a notable evolution. Sotorasib and adagrasib opens the prospect of achieving long-lasting responses that this oncogene is “untreatable,” demonstrating improved clinical efficacy compared to docetaxel and introducing diverse therapeutic options into clinical practice. However, the biological limitations of monotherapy and the inevitability of acquired resistance have forced an evolution toward advanced combination regimens—including immunotherapy, anti-EGFR drugs, or ADC conjugates. The simultaneous entry into clinical trials of highly optimized, next-generation molecules, such as divarasib, opens the prospect of achieving long-lasting responses while maintaining optimal quality of life for patients. In the coming years, as the final results of ongoing research programs are published, there will be a meaningful evolution in treatment guidelines, moving precision-targeted strategies to the very beginning of the therapeutic process and permanently improving the prognosis for this heterogeneous group of cancers.

## 6 Limitations

Interpretation of the results of this systematic review requires consideration of several key methodological and clinical limitations. The main factor determining the conclusiveness of the synthesis is that the evidence is based on a relatively narrow data set, limited to two multicenter Phase III registry programs—CodeBreaK 200 and KRYSTAL-12. Although both studies provide data from a representative cohort of patients, their open-label design increases the risk of attrition bias, which was only partially minimized by the use of BICR. Furthermore, the highly selective patient population (ECOG performance status 0–1, no significant comorbidities) significantly limits the ability to directly extrapolate efficacy metrics to the heterogeneous, real-world population of patients with advanced NSCLC (discrepancy between efficacy and effectiveness). Furthermore, from the perspective of current therapeutic standards, a significant limitation of the CodeBreaK 200 and KRYSTAL-12 trials was the selection of a suboptimal comparator in the control arm. In both studies, docetaxel monotherapy was used as the reference standard, whereas current ESMO and NCCN guidelines recommend a two-drug regimen—docetaxel in combination with an anti-angiogenic antibody (ramucirumab)—as the preferred and more effective second-line chemotherapy regimen in many regions. The lack of a direct comparison of KRAS^G12C^ inhibitors with this more potent regimen makes it impossible to unequivocally determine their actual advantage over optimal, current standard of care.

A key statistical challenge remains the complete lack of direct head-to-head comparative studies between sotorasib and adagrasib, which forces researchers to rely on indirect analyses burdened by population-level noise. Furthermore, the assessment of long-term benefits in terms of OS is hindered by an intra-study crossover effect. The crossover of a significant proportion of patients (as many as 26% in the CodeBreaK 200 trial) from the chemotherapy arm to the inhibitor arm following disease progression significantly obscured differences in OS and made it impossible to isolate a pure therapeutic signal without the use of complex mathematical models. In this context, although RWE analyses provide valuable data on the real-world efficacy of inhibitors outside the RCTs setting, differences in standards of care and molecular profiling across centers continue to pose a significant limitation to the homogeneity of these results.

From a clinical perspective, a significant factor that distorts the assessment of the safety profile is the omission of the actual dose intensity dynamics in reports. A high rate of dose reductions and treatment interruptions due to toxicity means that final PFS rates are correlated with the intention-to-treat (ITT) population rather than with actual drug exposure. Additionally, the lack of standardization in reporting overlapping toxicities following prior anti-PD-(L)1 immunotherapy—resulting from the absence of a defined washout period—makes it difficult to distinguish inhibitor-induced hepatotoxicity from late immune-related complications. Finally, it should be noted that limited access to comprehensive NGS molecular profiling in many clinical centers constitutes a systemic barrier that hinders the widespread implementation of these therapies in accordance with ESMO/NCCN guidelines, which impacts patients’ actual access to precision medicine. The analysis concludes with methodological inconsistencies in the assessment of intracranial activity—differences in eligibility criteria for patients with CNS metastases and the alternating use of the RECIST 1.1 and RANO-BM systems preclude the conduct of a reliable, quantitative comparative meta-analysis.

## 7 Conclusion

The incorporation of sotorasib and adagrasib into the standard of care for advanced NSCLC with the KRAS^G12C^ mutation represents a notable evolution in translational oncology. Overcoming the historical resistance of this oncogene to targeted therapy—a resistance that had persisted for over three decades—has demonstrated that covalent inhibition of the Switch-II pocket in the GDP-bound state provides a statistically and clinically significant advantage in terms of PFS and ORR compared to conventional second-line chemotherapy. Both molecules have demonstrated intracranial activity in clinical studies. However, differences in blood-brain barrier penetration and intracranial efficacy cannot be directly compared in the absence of head-to-head randomized trials.

The lack of a statistically significant prolongation of OS observed in registration trials should not be interpreted as low biological potency of the drugs, but rather as a direct consequence of the high rate of sequential crossover between treatment arms. This fact necessitates a redefinition of endpoints and statistical methodology in the design of upcoming clinical trials in the field of precision medicine.

However, the main structural barrier to sustained clinical success with monotherapy remains the limited duration of response, which is determined by the rapid development of acquired polyclonal resistance (through both on-target secondary mutations and the activation of off-target bypass pathways). Consequently, modern thoracic oncology is entering an era of advanced combination strategies. The future of care for patients with the KRAS^G12C^ mutation is likely to increasingly involve vertical inhibition of the MAPK axis, combinations with anti-EGFR antibodies, early combination with immune checkpoint inhibitors (as exemplified by the KRYSTAL-4 and KRYSTAL-7 programs), and synergy with modern ADCs. The simultaneous entry into clinical trials of next-generation molecules with optimized pharmacokinetics, such as divarasib may provide new therapeutic opportunities of achieving deeper and more durable anticancer responses while reducing organ toxicity, supporting the investigation of KRAS inhibitors at the earliest stages of radical treatment strategies for NSCLC.

## Data Availability

The original contributions presented in the study are included in the article/supplementary material. Further inquiries can be directed to the corresponding author.
